# Critical factors for the return-to-work process among people with affective disorders: Voices from two vocational approaches

**DOI:** 10.3233/WOR-182737

**Published:** 2018-06-27

**Authors:** Susann Porter, Annika Lexén, Suzanne Johanson, Ulrika Bejerholm

**Affiliations:** a Department of Health Sciences/Mental Health, Activity and Participation, Medical Faculty, Lund University, Lund, Sweden; b Department of Health Sciences/Mental Health and Mental Health Services Research, Medical Faculty, Lund University, Lund, Sweden

**Keywords:** Depression, bipolar disorder, supported employment, vocational rehabilitation

## Abstract

**BACKGROUND:**

Depression is among the major causes of disability with a negative impact on both daily life and work performance. Whilst depression is the primary cause of sick-leave and unemployment in today’s workplace there is a lack of knowledge of the needs of individuals with depression regarding their return-to-work (RTW) process.

**OBJECTIVE:**

To explore which factors are of critical importance for people suffering from depression and who also are unemployed in their RTW-process and to explore the impact of two vocational approaches on the service users’ experiences. The study included participants in two vocational rehabilitation approaches; Individual Enabling and Support (IES) and Traditional Vocational Rehabilitation (TVR).

**METHOD:**

Qualitative methods were applied to explore critical factors in the RTW-process. Individuals with affective disorders including depression and bipolar disorder were included.

**RESULTS:**

Three themes emerged as critical factors; *Experiencing hope and power, professionals*’ *positive attitudes, beliefs and behaviours,* and *employing a holistic perspective and integrating health and vocational service.*

**CONCLUSION:**

This study has demonstrated critical factors for the return-to-work process as experienced by persons with depression. To experience hope and power, to meet professionals that believe “you can work”, who use a person-centred and holistic service approach, are such factors necessary for gaining a real job. In particular, professionals in TVR need to embrace this understanding since their services were not experienced as including these elements.

## 1 Introduction

Mental health problems are the main cause of sick-leave and unemployment in today’s workplace [1, 2]. In Sweden, mental health illness is a fast-growing problem and is the leading cause of exclusion from the labour market [2]. Worldwide, the number of people suffering from depression is estimated at approximately 350 million [3] accounting for 4.3% of the global burden of disease. Depression is among the largest single cause of disability worldwide particularly among women with the longest periods of sick-leave [2, 4, 5]. It represents a substantial financial cost for society [6] since depression frequently starts in early life and is often recurring [3]. Depressive symptoms have a negative impact on daily life and work performance among those affected [3, 7, 8]. Nevertheless, research has shown that employment benefits these individuals in a positive way providing autonomy, well-being, reduced depression and increased social status [9, 10]. Individuals with bipolar disorder exhibit periods of elevated mood and periods of depression [11]. Globally it is estimated that 2.4% of individuals will be diagnosed with bipolar disorder during their lifetime [12] and a study undertaken in the USA showed the condition was associated with a high rate of unemployment of around 60% [13].

Individuals with affective disorders such as depression and bipolar disorder are studied together here because the depressive episodes individuals with bipolar disorder experience have a more disabling effect on work ability than the manic periods [14, 15]. People with affective disorders have received little research attention regarding evaluating effective vocational rehabilitation approaches while the evidence for people with psychosis, schizophrenia and similar conditions is on the other hand extensive [16, 17]. Research regarding individuals with affective disorders tends to focus on symptom reduction in healthcare settings separate from the work setting and vocational outcomes. As a consequence of this lack of understanding of effective RTW approaches there is a time and service gap between mental health services, vocational rehabilitation actors and employers. According to the review cited, individuals with affective disorders are a vulnerable group in this respect and their experience and need for support related to their RTW-process is neither evaluated nor fully understood [10]. In this study, we are using two approaches; the train-then-place model, which here is represented by the Traditional Vocational Rehabilitation (TVR) approach where the individuals first are submitted to prevocational training related to their disability and are then placed in vocational rehabilitation, then internship, and employment is the final step. The TVR approach follows an obligatory stepwise route and is not based on individuals’ preferences primarily. Secondly, the place-then-train model is seen in Individual Enabling Support (IES), a supported employment approach where individuals are promptly placed in employment according to their personal recovery and employment goals [18].

Previous research has primarily focused on evaluating separate interventions related to the mental health problem in certain steps of the RTW-process [10]. Therefore, the need to develop new interventions that address the difficulties individuals with depressive symptoms have with gaining and keeping employment has been recognized [10, 19–21]. As a response to this lack of effective interventions, two Scandinavian randomized trials were performed on the effectiveness of integrating supportive mental health strategies with the employment situation for people with affective disorders, with positive results [22, 23]. However, we have not yet learned what the service users themselves regard as the most critical factors for their process of job return, both with regard to new integrated vocational approaches and more traditional stepwise solutions. While randomized trials address certain predetermined outcomes, qualitative research is of great importance for the understanding of the intervention included in the trials.

Accordingly, we aim to develop an understanding of the service users’ own perspectives and experiences of factors they believe hinder or facilitate them throughout the RTW-process. This knowledge may benefit vocational service development and policy makers’ choices for future implementation. The aims of this study were as follows:

• To obtain the perspective of two vocational approaches; IES and TVR services which critical factors are of importance for persons with affective disorders (facilitates or hinders them) in their RTW-process.
• To discern the possible impact the two different vocational approaches, have on the service users’ experiences.

## 2 Method

This article describes a qualitative study that sought to explore critical factors in the RTW-process in people with affective disorders who were unemployed and participated in either a traditional or a IES supported employment approach to vocational rehabilitation for 12 months. The study is derived from a randomized controlled trial (RCT) designed to evaluate the effectiveness of supported employment adapted for people with affective disorders after 12-months. The study period commenced in December 2011 and ended in December 2014 [22].

### 2.1 Participants

In the RCT, participants were recruited from four geographically diverse outpatient settings in the County of Scania in southern Sweden [22]. Each participant was part of the intervention, IES or TVR, for 12-months. Participants were eligible (inclusion criteria) if they had: 1) been diagnosed with a depressive episode (ICD-10 F32), recurrent depression (F33.0, F33.1), bipolar disorder (F31), or (F30) includes depressive episode, as diagnosed by the team psychiatrist according to the International Classification of Diseases 10th edition [24], 2) were aged 18–63, 3) an ability to communicate in Swedish, 4) expressed interest in employment and in a RTW-process, 5) been unemployed for the preceding year. Exclusion criteria included severe drug/alcohol abuse and somatic illness or physical disability that impeded work.

**Fig.1:**
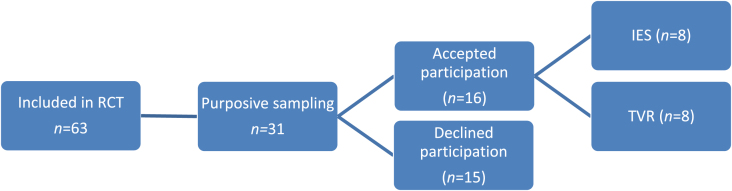
Recruitment process.

In this study, a purpose sampling of 63 participants who entered the RCT was used [25]. In addition to the RCT inclusion criteria, prior participation in the IES or TVR vocational rehabilitation for 12-months was a criterion. No adverse events or reported changes had been reported in relation to the RCT. Thus the only difference between the point when the participants entered their rehabilitation and the time for the current interviews concerned their age, since 12-months had passed. Before entering the RCT, they had received information about the project in the outpatient waiting room, daily newspaper, a website or through their case manager. Information meetings were then held by the research co-ordinator (SJ) at each outpatient unit, in which issues about the RCT, including the present interview data collection, could be raised [22]. All written information, including that in the consent form, had been approved by the Lund University Ethics Board, Lund, Sweden (Dnr 2011-544). All participants signed an informed written consent form. Out of the 63 participants, 31 individuals were purposely selected to the present study since diversity was sought for in relation to age, gender, service, i.e., Employment Specialist (IES) or handling officer (TVR), and outpatient unit. Two months previous to the time point for the interviews (10 months after enrolment), the participants were contacted according their preferences written in the consent form, i.e. by phone, e-mail or by letter. Sixteen participants accepted to take part (Fig. 1). Notably, all participants had some experience of RTW activities according to TVR prior to enrolment in the RCT. During their structured participation in the current services, however, experiences of two vocational approaches were possible to describe for the participants in this study. If support was still needed, participants could return to the TVR services after the project had ended.

### 2.2 Individual Enabling and Support (IES)

The IES approach was developed for individuals with affective disorders and is guided by an Employment Specialist who works closely with the participant in relation to the outpatient team, family, Social Insurance Agency (SIA), Public Employment Service (PES), and employers. The Employment Specialists receive a three-week long training in motivational interviewing from a certified motivational interviewer, cognitive strategies from a Cognitive Behavioural Therapy psychologist, Time-for-Work strategies from an Occupational Therapist and training in supported employment from Supported Employment specialists, reinforced through face-to-face supervision. The Employment Specialists professional role requires high quality, empathetic counselling corresponding to ten IES principles, which can be carried out in any order. These are: 1) handling change and developing motivational and cognitive strategies related to RTW, 2) integration of IES with mental health treatment, 3) having a time-use pattern that supports work-life balance, 4) eligibility based on client choice, 5) competitive employment as a primary goal, 6) job search based on personal preferences, 7) rapid job search, 8) benefit counselling (SIA/PES) at an early stage, 9) ongoing support and work accommodations as needed and 10) systematic recruitment and quality engagement with employers [22].

### 2.3 Traditional Vocational Rehabilitation (TVR)

The TVR process is regulated by the social benefit and unemployment security system. Various professionals in several settings and organizations, e.g. healthcare, municipality, SIA and PES, are part of the TVR process. The service is individualized to a lesser extent and is delivered in several assessment stages that are regulated by the organizations involved. The first step includes reducing symptoms and increasing work ability at a mental health service (1 hour per week). The following step involves assessment of the individual at 50% work capacity (10–20 hours per week) and is performed by the SIA and PES. If work capacity is not met, the individual is encouraged to enter the third step with prevocational activities at the municipality. This is regulated by law at 5–20 hours per week. The final step is vocational training during internship placements (20–40 hours per week), and these placements can lead to employment positions through the PES [18, 26].

### 2.4 Data collection

In-depth interviews (IDI) were performed with participants between January and July 2014. IDI is a loosely structured individual interview method performed on a small number of participants [27]. This interview technique is ideal for exploring and evaluating thoughts on participants’ intervention processes and outcomes and the impact their involvement had on the perception of themselves [25, 27]. It requires concentration and focus and therefore trustful interaction between interviewer and interviewee (participant) is essential for the quality of information generated. The research questions were developed by the RCT research team [22]. In previous RCT and implementation research it had been identified that there seemed to be different ways of approaching and providing support to people with mental health problems in the RTW-process [18, 22, 28]. Furthermore, it is crucial to use qualitative methodology to understand long lasting complex interventions [29]. Since no previous qualitative research has been performed on the topic, the questions were open and concerned exploring experiences related to the RTW-process. This is in contrast to a semi-structured questionnaire with more detailed and specific questions [30]. Research questions were developed in relation the experiences of own and others’ perspectives on work and mental health and support provided in relation to RTW process. Examples of open questions used in this study were related to work: *Can you describe your aspiration to work? How do you perceive yourself in relation to work? Does anyone or anything hinder you from applying for a job/to work?* Related to the social environment in terms of family, vocational and health professionals: *Can you tell about the support you experience from others in your RTW-process?* Questions regarding various welfare organizations involved: *Can you describe how welfare actors support your aspiration to work?* Additional probing questions were asked as needed: *Can you give me an example? Could you elaborate on that idea?* One researcher (AL), a skilled interviewer in research with considerable experience of working with and supporting people with affective disorders in clinical practice, conducted all interviews. AL had no previous role in the research team and no previous connections between the interviewer and interviewees existed. In 2013, prior to performing the interviews, the interviewer undertook training in qualitative research methods and interview techniques. Since the questions were open, only few, and not standardized into a semi-structured questionnaire, it was not meaningful to test the questions or themes in a strict manner. Therefore, the interview situation was tried out twice prior interviews in the present trial and discussed between AL and UB in relation to the data elicited and aim of the study. The interviews, which lasted for approximately one hour, took place at an outpatient unit and were digitally recorded. All materials were stored on a USB drive held securely in a locked cupboard at Lund University with access restricted to researchers involved in the study.

### 2.5 Data analysis

The interview material was transcribed verbatim by an independent research assistant. The manifest content analysis according to Graneheim and Lundman was used as a method [31]. The reason for using a manifest content analysis was to stay close to the spoken materials that were communicated during the interviews. As a first step, each interview was analysed and meaning units that corresponded to the research aim were identified and tabulated. Each meaning unit was then condensed while conserving the core of words used by the participant. The first author (SP) conducted this passage of the content analysis and the last author (UB) and independent research assisted this process. In the final step, a code was abstracted from the developed logic of words chosen. A code can be understood as fragments of meaningful information. The development of a coding framework was an iterative process among the authors. Once formulated, the codes were compared to each other, read and re-read, for each interview separately and for all together. Similar codes formed categories and sub-categories. Both positive and negative responses could represent the same category. Additionally, to ensure the credibility, the first author (SP) performed text verification following the transcription by replaying the conversation audio files and validating against the transcribed scripts. This additional step was taken to ensure that the transcribed interviews were sufficiently accurate [30]. Finally, the coding categories were combined into themes that corresponded to the more inductive phase of the analysis. To support the secondary aim of the study regarding experience of the users of the two RTW approaches, within and across analysis were performed [32]. This information is integrated within the results.

## 3 Results

A total of 31 individuals fulfilled the inclusion criteria were asked to participate in the study. Of those 16 (*n* = 16) gave informed consent for study participation. The characteristics of those included are presented in Table 1. The individuals in the IES comprised four men and four women with a mean of 38.8 years (range 24–54), and the TVR two men and six women with a mean of 44.9 years (range 22–57). The occupational status differed between the participants in IES and TVR regarding employment (*n* = 5 vs *n* = 0), internship (*n* = 2 vs *n* = 1), mainstream education (*n* = 1 vs *n* = 1) and prevocational training/activities (*n* = 0 vs *n* = 6). Not all information was obtained from all participants for unknown reasons. The 15 participants from the RCT (6 IES and 9 TVR) who declined participation comprised 3 men and 12 women with a mean of 40.9 years (range 20–57). The main reason for non-participation was not answering the phone when contacted (*n* = 11). Specific individual reasons (*n* = 4) were; did not want to because of the private situation, just had a baby, moved abroad and difficulty being absent from work.

**Table 1:** Demographic and clinical characteristics of the participants (*n* = 16)

Characteristics	Participants
Sex
Female/Male	10/6
Age in years
Mean, SD (Range)	39.3, 12.5 (22–58)
Country of origin
Sweden	14
France/Vietnam	1/1
Civil status
Married/not married	5/8
Divorced	3
Living situation and children
Cohabiting/living alone	9/7
Have children yes/no (*n* = 13)	8/5
Educational level
Middle school <16	2
Upper secondary >16	6
College/university >18	8
Occupational status
Employment	5
Internship	3
Mainstream education	2
Prevocational training/activities	6
Clinical characteristic
Depression (female/male)	11(5/6)
Bipolar (female/male)	5(5/0)
Age in years at first contact with
psychiatry mean (range)	28 (12–49)

**Table 2:** Critical factors for the RTW-process

Theme	Categories
Experiencing hope and power	Prejudice and lack of knowledge about mental health problems
	Dilemma of disclosing mental health problems
	Self and identity roles
	The family’s participation
	Low self-esteem, little belief in changes and support along with low self-efficacy
Professionals’ positive attitudes, belief and behaviour	A genuine interest and engagement from those who provide support and treatment
	Gender impact on treatment
	To understand the individual needs and conditions in the vocational rehabilitation
	Letting the individual’s needs lead the intervention
Employing a holistic perspective and integrating mental health and vocational services	Need of coordination and person-centered service
	Bureaucratic structure hinders vocational rehabilitation
	Traditional vocational rehabilitation is unable to help the individual
	Advantages of the place-then-train IES vocational approach

Three themes emerged related to the aims of the study as shown in Table 2. *Experiencing hope and power* was perceived by most of the participants, regardless of the approach, as an important facilitating factor in their RTW-process. They had own experience of, or were worried about, exposure to prejudice in interaction with others at work e.g. colleagues and managers regarding disclosure. Their self-efficacy was diminished as a majority expressed a lack of confidence regarding returning to work. *Professionals*’ *positive attitudes, belief and behaviour* were expressed as crucial factors. In this study, the RTW professionals could be seen as either a facilitating or a hindering factor depending on how they interacted with the individual. *Employing a holistic perspective and integrating health and vocational service* was expressed regarding the need of a coordinated person-centred service with the same professional involved in the rehabilitation with the individual. Bureaucratic structures were seen as a hindering factor when seen as inflexible and not adapted to the individuals’ need. The two Employment Specialists were experienced as facilitators, making the participants feel supported, understood and listened to regarding their individual desires and needs. On the other hand, the feedback from the TVR-participants attending the same traditional prevocational and vocational training and support regarding support was expressed as mainly negative; a feeling of lack of engagement with the individual not feeling important or not having their needs in focus.

### 3.1 Experiencing hope and power

This theme concerns the importance of *experiencing hope and power* in the RTW-process. For example, the following elements formed parts of the theme; how participants perceived others’ views about mental health problems in relation to themselves, disclosure of ones’ problems, roles of importance in their life and view of themselves in terms of self and identity roles and self-efficacy. All participants were represented extensively regardless of the vocational approach.

#### 3.1.1 Prejudice and lack of knowledge about mental health problems

Most of the participants experienced that others displayed prejudice toward people with mental health problems; this was extensively expressed during the interviews and experienced in a negative way. Participants expressed their concerns and fears of being socially excluded and disadvantaged at work if the employer knew about their illness. To be worried about being alienated, not treated fairly or feeling exposed were thus some feelings expressed. A fear of prejudice and lack of knowledge were given reasons not to disclose their illness to others or hesitating to divulge due to the change, or worry about change, in how the person felt they would be perceived. Sometimes the participants expressed a worry about disclosing their mental illness even though they had not yet told anyone. No discrepancy between the two RTW approaches emerged. A woman of 39-years of age (IES) talking about her concerns of prejudice;

“*I think lots of times people are* … *very scared of people with mental health problems.*”

Another woman, 47-years old (TVR), explained;

“*I think that it would have been easier if I have had a nicer (with irony) diagnosis like cancer. Then you get much more sympathy because then you have not brought it on yourself, but have just become ill.*”

#### 3.1.2 Dilemma of disclosing mental health problems

The category about mental health disclosure represents the dilemma of whether to tell others about ones’ mental health problems. Experiences were divided among the participants in terms of positive and negative experiences however bad experiences were in the majority. Outcomes from disclosure were revealed as; leading to degrading work tasks, more pressure from the manager, a feeling of exposure or a worry about being judged as “crazy”. Positive experiences from disclosing the mental health problems to the employer could be described as the employer gained greater awareness of work capacity, could have a better understanding and recognition of the individuals’ mental health problems and the employer could set realistic goals matching the individuals’ capabilities to work. Furthermore, employers were given insight as to why an individual sometimes felt bad. Some IES-participants expressed how the Employment Specialists supported them in their decision regarding disclosure and felt they could be open and honest in relation with them. Consequently, IES-participants were more positive than the TVR-participants because of this decision support. A woman 38-years old (IES), was given disclosure decision support prior to a work interview from the Employment Specialist;

“*At the first interview, I didn’t do that, I didn’t tell anything about it and that was something I discussed with X (Employment Specialist name) and her approach was that - Why? You don’t have to sit there and tell you have diabetes. There is no such thing that you have to tell.*”

#### 3.1.3 Self and identity roles

This category constituted the roles the participants had in their lives and what they meant to them. It became clear that the work and family roles were important for self-identity, self-confirmation and self-perception. The work role was of great importance for how they perceived themselves. It gave them confirmation, a sense of identity, meaning and a feeling of being competent. Without the work role, their view of self and identity were sometimes expressed as lost. Woman, 39-years old (IES), talking about the importance of having a work role;

“*To feel like I am part of a context, that I am part of society. When I was unemployed I felt outside, I felt like the earth was spinning without me being part of it. When I had a job, I felt I was also a part of the spinning, then it was totally okay.*”

Most participants also talked about the family role, specifically how families contribute to a feeling of being safe and confirmed. In relation to this role, the participants wanted to be perceived as someone who had positive qualities, e.g. were competent and knowledgeable and a positive person. No discrepancy between the IES and TVR-participants could be identified regarding the category of *self and identity roles*.

#### 3.1.4 The family’s participation

Whilst the family role was seen as significant for the participants’ RTW-process this stands in contradiction to the actual experience presented within this category. The participants often felt a lack of understanding and support from their partner or family regarding their diagnosis and the challenges in their life following the illness. There was no difference between the IES or TVR-participants in this regard. Woman, 56-years old (TVR) describes her husband’s lack of support regarding her problems;

“*My husband has the most difficulty to understand I would say, to understand or wanting to understand* … *my needs.*”

A 26-year old man (IES) expressed how his stepfather constantly nagged at him;

“*You have to do something*! *I’m like okay, yes. And then I really try, but it doesn’t work.*”

#### 3.1.5 Low self-esteem, little belief in changes and support along with low self-efficacy

The participants regardless of the RTW approach, experienced, or had experienced in the past, low self-esteem and little belief in the future in relation to their RTW. Half of the TVR-participants were also worried about relapsing in their illness. In contrast, although the IES-participants had experienced little hope in the past, they now expressed hope for the future through their new experience of working with the Employment Specialist. The support can be seen as a change in how they perceived their prospects to find a job and their self-esteem regarding returning to work. A woman, 38-years old (IES), talking about the Employment Specialist in the context of believing in her ability to work;

“*This was someone who actually believed that I could start working again. She was very supportive, very like* … *Wow, you have a great CV and there is nothing that can stop you from getting back to the labour market. Thus, there were no sighs or moaning or something like that, just positive energy.*”

### 3.2 Professionals’ positive attitudes, belief and behaviour

This theme reflects the discrepancies between TVR and IES-participants in their RTW-process. According to the IES-participants, the Employment Specialist was a key facilitating factor. The specialists were committed to support them, respected them and their situation, and had a genuine interest in supporting them back to work. The TVR-participants on the other hand expressed more negative attitudes towards their vocational rehabilitation in general and the professionals in contact with them. The professionals showed a lack of commitment and empathy, they did not prioritize the TVR-participants in their RTW-process and misunderstood critical situations like the individuals’ mental health problems. A woman, 47-years old (TVR), talked about a time when a handling officer from the SIA called to inform her that she was not ill enough to receive sick-leave benefits and get vocational support. The woman responded;

“*I hope she can sleep really well at night because, because I have tried to take my life several times.*”

#### 3.2.1 A genuine interest and engagement from those who provide support and treatment

In this category, a notable difference in the perception of vocational rehabilitation support among the participants was shown. A genuine interest and commitment from those providing RTW support was thus a critical factor for a successful RTW. The IES-participants, who had experienced TVR prior to their participation in IES, mentioned a cold and hard attitude at the PES and a lack of engagement among professionals in the TVR approach in general as one 26-year old women (IES) expressed the following when talking about her PES handling officer;

“*The handling officer showed no understanding. He wanted to see result so he was very cold and hard in that way.*”

No such experiences were expressed in relation to the Employment Specialist, who functioned as a facilitator of the RTW-process for the IES-participants. They were viewed as being very positive and being professionals who showed respect. The participants in the TVR approach also reported bad experiences, such as a lack of commitment from professionals at the SIA, a lack of understanding and empathy, and a feeling of indifference from the professionals at the PES.

#### 3.2.2 Gender impact on treatment

Five of the participants believed that gender impacted on how they were being treated in their RTW- process. Opinions from women were; a feeling that women are more frequently long-term unemployed than men, the diagnosis was downplayed because of (her) gender and one participant felt that she was less listened to due to being a woman. Mens’ opinions of how their gender affected treatment were a feeling of higher demands on a man to work and that men with depression are more vulnerable as they can be perceived as weak. A man 29-years old (IES) talking about how men can be more exposed than women when having depression;

“*No, it’s not that easy actually, it’s rather tough when you are a bit different being a man.*”

Another participant, a woman 58-years old (TVR), expressed how she felt that professionals did not listen to women in the same way as they listened to men;

“*Yes, sometimes I think it’s harder to make yourself heard. I think that when I talk to handling officers at the PES, if I had been a man, I think they would have listened more.*”

Despite these impressions from some participants no discrepancy could be identified between the IES and TVR participants regarding the category *gender impact on treatment.*

#### 3.2.3 To understand the individuals’ needs and conditions in the vocational rehabilitation

For IES-participants support was provided mainly by the Employment Specialist, who functioned as a representative in relation to meetings and appointments with different actors in the RTW-process. Two TVR-participants expressed that their administrator at PES acted in a similar way, i.e. was supportive and understanding for their needs. However, most of the TVR-participants did not feel they had support in their RTW-process and perceived feelings of hopelessness. Furthermore, some IES-participants explained how the Employment Specialist could reduce stress and provide relief. They experienced the support to be flexible and according to their needs and described being treated with honesty and respect. When needed the Employment Specialist provided support in relation to authorities and employers. This was viewed as positive since it helped to avoid misunderstandings about ones’ problems. They also supported participants’ individual needs in relation to job interviews. A common perception among all participants was that there was a lack of understanding about depression and support strategies among TVR professionals as well as employers. There were five participants who expressed concerns about their financial situation. They felt isolated due their financial situation and their personal economy was perceived as a barrier to participation in life. However, two IES-participants turned this situation around with support from their Employment Specialist. Man, 26-years old (IES) sounding pleased when he talked about how the Employment Specialist helped his financial situation when talking to the PES and the employer so he could get a job with a wage subsidy salary;

“*She (Employment Specialist) arranged this wage subsidy, the one I have now. She fixed it* … *she talked to the unemployment centre and talked to the employer.*”

#### 3.2.4 Letting the individual’s needs lead the intervention

This category included facilitating factors regarding the individuals’ wishes for their vocational rehabilitation e.g. the importance of personalizing the vocational rehabilitation to adjust to the individual’s needs and the importance of having the same professional involved in the process. These factors were expressed by most of participants to be critical for their rehabilitation. Repeatedly, the IES-participants expressed that their Employment Specialist worked in their best interest, knew them well, and worked according to their wishes and conditions. According to some of the IES-participants’ experiences, they believed that obligatory prevocational training according to the train-then-place approach of TVR was pointless e.g., “*meaningless to show up there* and *having a work training place doesn’t lead anywhere.*” Furthermore, some participants felt it was unnecessary since they had substantial work experience already. One IES-participant experienced an internship placement as being positive however as it was a way of getting back to work without having too much pressure. A 47-year-old woman (TVR), experienced good, positive and individual support from her administrator at PES when receiving an internship of one day per week, working in a field she knew she was competent in;

“ … *and so it all changed because I’m an expert in this, I am very good. Completely safe, it’s nothing that can stress me.*”

### 3.3 Employing a holistic perspective and integrating mental health and vocational services

This theme corresponds to a variety of hindering factors commonly expressed. The lack of coordination of individual support in the participants’ RTW-process and a change, and amount of, professionals were two such factors. Participation in the IES was on the other hand viewed as a strong facilitator for the RTW-process and the person-centred service provided by the Employment Specialist was highlighted as critical.

#### 3.3.1 Need of coordination and a person-centred service

Two restrictive factors expressed by most participants were the lack of coordination and not receiving a person-centred service throughout the RTW-process. All participants experienced that a substantial amount of contact with different authorities was common in the train-then-place TVR approach. The IES-participants had this experience prior to their IES-participation and the overall experience was negative. The traditional rehabilitation system was seen as fragmented, inflexible and irresponsive to the individuals’ needs. Professionals were experienced as being unengaged and lacking coordination strategies among different central actors. The change of professionals can be seen as a hindering factor as it was experienced as stressful and impairing the development of relationship and trust. For example, a male participant described how he had seen around six different PES handling officers, which he experienced as stressful. Another expressed how she fell through the cracks between organisations and yet another that her needs were not taken seriously. No differences between the two groups could be discerned.

#### 3.3.2 Bureaucratic structure hinders vocational rehabilitation

The bureaucratic structure embedded in the regulation of the different vocational services in Sweden was seen as a hindering factor for the participants’ RTW-process. The TVR services provided by the authorities were inflexible and caused frustration since the support was not adapted to individual needs. Experiences of bureaucratic structures were regularly described by participants. One participant, a woman 58-years old (TVR), initiated an internship placement by herself that suited her needs. Unfortunately, this individual initiative was not appropriate according to the PES professional;

“*I fixed an internship placement myself that I knew was going to work out because I know myself but they didn’t accept that. Instead they gave me another placement in a company that went bankrupt.*”

#### 3.3.3 Traditional vocational rehabilitation is unable to help the individual

All participants expressed how the prevocational assessment and training as seen in the TVR approach was an inadequate support for them in their RTW-process. The majority expressed a lack of support and a feeling of hopelessness in this kind of vocational rehabilitation. Three individuals claimed their depression became worse or had experienced relapse due to the negative outlook of the TVR vocational process. A 26-year old woman (IES) recounted how she was perceived in contact with PES administrators from previous experience in TVR;

“*Thus, they behave like you were too lazy to fix a job and that you actually have to pull yourself together.*”

#### 3.3.4 Advantages of the place-then-train IES vocational approach

Naturally, in this category only IES-participants were respondents, as the TVR-participants had no experience of this approach. The Employment Specialist who directly worked with the participants offered individually tailored support which helped to build up their self-esteem and to regain self-belief in their ability to work again. The Employment Specialist had a holistic view and supported the participants in different issues such as administrations tasks regarding different authorities. They also acted as someone the participants could easily reach and talk with. No negative feedback was mentioned and the approach was experienced positively. A 55-year old man was talking about the importance of having the Employment Specialist to talk to;

“*This IES project then, if you take this Employment Specialist part, which has been of great help for me because it has given me someone to talk to. We have talked almost every week, sometimes more than once a week which has been of great help for me.*”

## 4 Discussion

This study focused on how personal, environmental and structural factors facilitate or hinder the RTW-process as experienced by the service users when having depression and being in unemployment. The results highlight the importance of a person-centred approach adapted to and addressing each individual’s need for RTW support. The importance of the RTW professional to give hope, power and support were seen as critical facilitation factors in this study along with positive attitudes and belief in the individuals’ potential together with a holistic perspective surrounding the whole process. The bureaucratic structures experienced in the *train-then-place-model* TVR [18, 33] were perceived as a hindering mechanism. Conversely the *place-then-train model*, as seen in the IES approach, was experienced as a significant facilitating factor delivered in a personal-centred manner. A successful RTW-process requires the supporting professionals to have a broad knowledge and belief in the individuals’ abilities to resume working life, an outcome the individuals themselves often can struggle to see as achievable. For this to be accomplished, mental health literacy in vocational rehabilitation needs to be increased among professionals delivering the RTW-process [34].

### 4.1 Others and own view of self-internalized stigma

Participants in this study expressed to a high degree anxiety concerning stigmatizing of their mental problems and faced dilemmas regarding disclosure to colleagues and employers. These findings are in line with previous qualitative research by Gladman and Waghorn [35]. They found disclosure of mental health status in the work setting to be a critical issue that could lead to discrimination, of not being hired, or even, being fired [35]. Internalized stigma is common among individuals with mental health problems [36] and previous research has also shown discrimination at the workplace was both expected and experienced if one disclosed mental health problems to the employer [35, 37–39]. Better collaboration is needed between employees suffering from depression and their manager to enhance work performance [40]. Whilst it was occasionally seen as a positive step to disclose, the majority felt this would result in negative consequences. However, a distinction could be seen between the two RTW approaches. Even though participants in the IES experienced stigmatizing of their mental problems, they expressed more positive feelings around disclosure when the Employment Specialist supported this within the RTW-process, in accordance with a previous RCT on disclosure of mental health to employers [21]. Furthermore, since the work role was communicated as one of the most important roles in their lives, the risk of a negative outcome when disclosing had high potential impact. This has been seen in previous research such as Hamilton [41] who observed individuals with mental health problems experienced to large extent discrimination when searching for or retaining a job. A systematic review where employers expressed their feelings about hiring a candidate with depression showed this not just to be an internalized stigma. These employers considered depressed individuals to be less likely to be appointed and were less positive in terms of hiring a person with depression than a person with a physical disability [37]. In our study participants with depression showed a great concern and fear of being excluded in the social context and of having to face disadvantages at work if their problems were known. One could ask why individuals with depression feel stigmatized in the work setting but also in other social contexts including within their own family, when the family role is also seen as so important for them? Many reasons could lie behind the lack of understanding in the family context. Diminished participation in family activities, a lack of contribution to the work of the household (leaving the partner with the majority of chores) and not contributing to the household economy could lead to stress and a lack of understanding regarding the underlying problems. When reviewing the interview material, it was evident that lack of support from the family was a great sorrow for those who experienced it. This result agrees with the findings of a previous qualitative study (*n* = 537) also showing discrimination in the family to be common due to having a mental illness as seen as a lack of belief in the illness to be genuine [41]. This burden is brought to the RTW-process and it is important to consider when working alongside these individuals. This knowledge of how the participants in this study viewed themselves and their feeling of stigmatization and conversely of hope and power for their RTW are critical factors. Including these findings could improve the RTW intervention in a material way among mental health professionals.

Conceivably there is a macro-effect of lack of knowledge and understanding about mental health in society and with increased awareness, prejudice could become less prevalent, as addressed in the literature of mental health literacy [34, 42]. Another reason closer to the circumstances of the individual could be the time and service gap between the mental health care and the vocational rehabilitation as seen in the TVR approach. This could impact on the depression in a negative way and prolong or delay a positive outcome [7] when in contemporary society work is a measure of value and success. Lower occupational function can lead to social exclusion [1]. Hence when an individual has a depression and is also unemployed it could increase the risk of being stigmatized in a double sense.

### 4.2 Professionals are a decisive factor for a successful RTW-process

Most of the TVR-participants communicated deficiencies in their RTW-process such as the rehabilitation not being centred on their needs. They conveyed a lack of understanding, empathy and belief from the professionals, which was seen as significantly negative. This has also been observed by Lammerts [43] who identified professionals’ difficulties to find work solutions like competitive jobs most probably because of a lack of belief in positive RTW outcomes. We find it notable that professionals in this field did not appear to demonstrate supplementary knowledge about depression to be better able to support the service users’ needs to reach a successful outcome. When working with individuals suffering from depression who are returning to work, this lack of engagement, faith and belief in the individual are seen as fundamental ingredients. The individuals already doubt to a great extent their own ability to work and if the professional does not support the opposite view a successful outcome is less likely. This has also been recognized in a previous study to be a concern, when clients with mental health problems described too little support from vocational rehabilitation agencies and no consideration of their personal wishes [43]. Having an individual approach in the RTW-process of individuals with depression is of importance according to Andersen [44]. In contrast, individuals participating in IES conveyed a much more positive experience. They felt their Employment Specialist supported them and wanted to help. Participants believed their rehabilitation was personalized by considering their wishes and interests and that they were empowered to make their own decisions. An IES trial by Bejerholm [22] shows the IES approach has a more successful employment outcome along with a reduced depression and increases quality of life compared to the TVR approach.

### 4.3 A holistic perspective throughout the RTW-process

Most of the TVR-participants described negative experiences including a lack of coordinated, person-centred approaches and overly bureaucratic structures, which were also identified by Lammerts [43]. In our study, the IES-participants, who all had some previous experience of TVR, expressed a switch from negative to positive RTW experiences. Their individual needs were in focus, not only their diagnoses and problems and the outcome was to secure a real job, not prevocational, work training or internships, which could be seen as degrading. A previous study also showed the importance for these individuals of a real job rather than an internship [45]. Despite depression being among the most prevalent illnesses in the western world [43] it is discouraging that experiences displaying such a lack of knowledge and understanding for individuals’ needs exist. As Chisholm [6] reports, this not only causes the individual to suffer but also society as a whole, which forgoes the productive potential of individuals not actively participating in the workforce. The financial investment in effective treatment of depression leading to employment would according to this analysis deliver a significant positive return for the economy. Whether this is simply a lack of mental health literacy among the professionals involved or other reasons that impact the service delivery requires further research.

Joyce [10] showed in a meta-review that to promote functional recovery after depression requires integrated approaches that include functional goals such as RTW exposures as part of the treatment. Symptom focused interventions such as medication and psychological treatment showed limited effect on the work-related outcome and to withhold the RTW process until the individual is symptom free did not show better outcomes. On the contrary, the RCT of which this study is part [22] showed a higher employment rate for individuals with affective disorder when using the IES approach and placed in employment promptly, as reflected in this study’s demographics.

### 4.4 Methodological considerations

In this study, a purpose sampling was used in terms of diversity regarding age, gender, service, Employment Specialist or handling officer and outpatient unit providing a broad view of service users’ experience. This technique increases the trustworthiness in qualitative research and increases the possibility to “shed light on the research question from a variety of aspects” as it broadens the possibility of having participants of different ages, gender, diagnoses etcetera [25]. IDI was a method that worked well in gaining detailed information of the participants’ thoughts and experiences. The interviewer had long clinical experience working with individuals with depression and used an effective interview technique [27]. It could be argued that the sample size of this study was insufficient to draw strong conclusions. However, it was never the purpose of this qualitative research to generalize. Instead the focus has been to show the broad opinion and experiences from these specific individuals. The use of quotations was limited to those expressing content that increased the credibility of the analysis [31] and included to give the reader a deeper understanding. Of 31 possible participants, 15 chose not to participate. It is not possible to fully understand the causes however recruiting participants is a common problem with similar participants. In a qualitative study of individuals on long-term sick-leave with depression and stress conducted in Denmark, only 20% agreed to participate [44]. In our study, even with a dropout of 15 participants it was clear saturation was reached when consistent views of the experience emerged [27].

## 5 Conclusion

The aim of this qualitative study was to develop an understanding of the service users’ own perspective and experience of factors they believe hindered or facilitated them in their RTW-process. The result shows critical factors to be: having the experience of hope and power, being supported by professionals who show positive attitudes, belief and behaviours and work according to a holistic approach, in a person-centred service in which the outcome concerns a real job. Mental health problems have historically faced a lack of understanding that persists even today [46]. However, this study has provided new and broader knowledge in capturing and understanding individual’s needs in the RTW-process through the participants’ own voices, knowledge that is compatible with previous findings in a case study of severe mental illness [47]. To our knowledge, this is the first qualitative study done on people with affective disorders focusing on depression in unemployment capturing the individuals’ thoughts and experiences of two vocational approaches simultaneously. The result highlights important critical factors for a successful RTW-process and is of great importance for professionals working clinically with these individuals that can be implemented in the clinical work and benefit individuals with depression in their RTW-process. For this change to occur other key stakeholders such as politicians also need a greater understanding of the role of these critical factors in delivering successful RTW outcomes in order to establish the necessary regulatory and budgetary frameworks.

## Conflict of interest

None to report.
